# Pregabalin Toxicity in Kidney Failure: A Medication Error With Neurological Consequences

**DOI:** 10.7759/cureus.100611

**Published:** 2026-01-02

**Authors:** Adam G Selway, Shea Roffey, Anthony F Brown, Maddison Taylor, Peter Del Mar

**Affiliations:** 1 Faculty of Medicine, University of Queensland, Brisbane, AUS; 2 Emergency and Trauma Centre, Royal Brisbane and Women's Hospital, Brisbane, AUS; 3 Department of Kidney and Transplant Services, Princess Alexandra Hospital, Brisbane, AUS

**Keywords:** emergency medicine, emergency medicine pharmacist, medication error, pregabalin toxicity, renal failure

## Abstract

We present a case of pregabalin toxicity in a 50-year-old dialysis-dependent female, who presented with a one-day history of progressive weakness, lethargy, and gait disturbance. They were successfully treated with haemodialysis. The case is notable for the atypical toxidrome seen in kidney failure, which occurred after a community pharmacy error led to a single twelvefold-increased dose. It also demonstrates the role of the emergency department pharmacist, who was crucial in identifying the medication error. The report emphasises the importance of careful medication reconciliation, especially in patients with kidney failure. It also highlights the importance of considering gabapentinoid toxicity in any patient who presents with new, unexplained neurological symptoms.

## Introduction

Pregabalin is a gabapentinoid that is frequently prescribed to dialysis patients to treat symptoms such as neuropathic pain, restless legs, and uraemic itch. As pregabalin excretion is proportional to creatinine clearance, dose adjustment is required for patients with reduced renal function [1]. There is therefore a high risk of accumulation and toxicity in these patients should a medication error occur. Having pharmacists embedded in the emergency department (ED) has been shown to reduce medication errors and improve completeness and accuracy of medication histories [2]. Their role is therefore particularly valuable in the management of high-risk patients, such as those with kidney failure. Gabapentinoid toxicity has been infrequently reported in kidney failure patients, and given its low volume of distribution, dialysis is an effective treatment [1,3,4]. Neurological symptoms of accumulation in kidney failure can present with myoclonus and drowsiness. Other symptoms, such as aphasia, have been less frequently reported [3-5]. We make a report of a community pharmacy dispensing error, which resulted in a patient with kidney failure developing significant neurological symptoms after a single increased dose of pregabalin. This was rapidly identified by the ED pharmacist and successfully treated with dialysis. The report highlights the critical role of the ED pharmacist and the need to consider gabapentinoid toxicity in a patient with kidney failure presenting with new, unexplained neurological symptoms.

## Case presentation

A 50-year-old dialysis-dependent female, with kidney failure secondary to hypertension and type 2 diabetes mellitus, presented to the ED with a one-day history of progressive generalized weakness, lethargy, and gait disturbance. Her other medical history included rheumatic heart disease with severe aortic regurgitation and ischaemic heart disease.

On examination, the patient was hypersomnolent but oriented, responding slowly but appropriately. Her vital signs were normal. Examination of limb power was highly variable, with intermittent ability to generate 5/5 power throughout, then abrupt giving way. Involuntary upper limb movements and bidirectional nystagmus were also noted, while the cranial nerve examination was otherwise unremarkable. The patient complained of extreme tiredness and generalised weakness that began that day, a day she had been due to dialyse. When asked directly, she admitted to some vague neck pain but denied any other symptoms. She also denied intentional overdose, recreational drugs, or alcohol.

**Figure 1:**
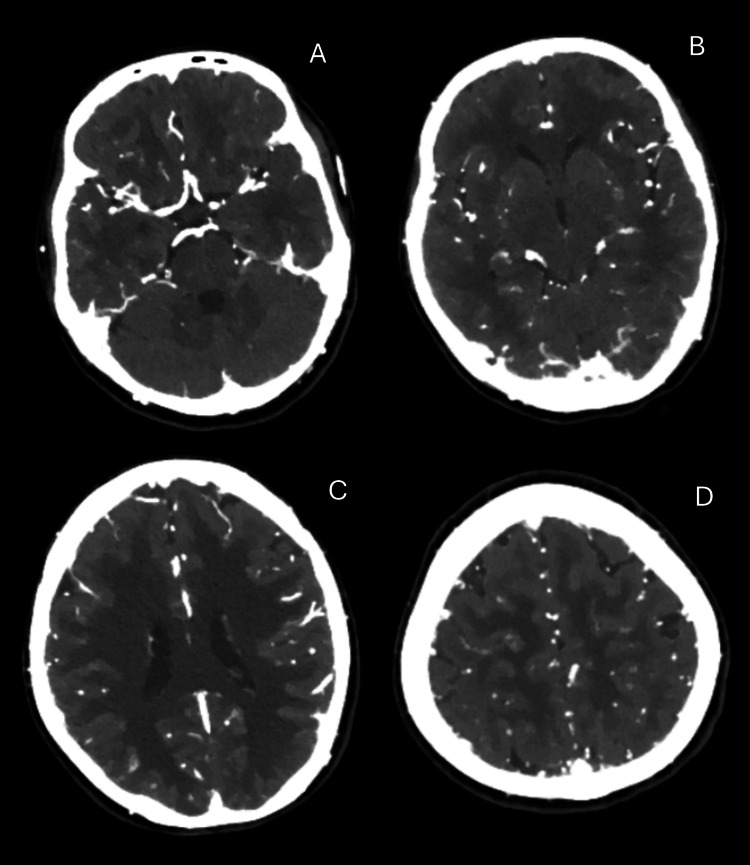
CT head and neck angiogram A-D: transverse planes of a CT head and neck angiogram, which were unremarkable and demonstrated no structural or vascular cause for the presentation

Initial investigations looking for common metabolic or infectious causes revealed only a mild metabolic acidosis on venous blood gas. Laboratory blood tests showed an expectedly elevated urea, mildly raised potassium, and a chronically elevated and stable troponin. A mild neutrophilia was also noted (Table 1). None of these investigations was felt to be abnormal enough to represent a clear cause of her symptoms. A non-contrast CT of the brain, head, and neck angiogram, to rule out structural or vascular causes, was unremarkable (Figure 1A-D). 

**Table 1:** Results from venous blood gas, initial laboratory investigations and repeat troponin, demonstrating no clear cause for the symptoms

Test	Result	Reference range
pH	7.32	7.32-7.43
Bicarbonate (HCO_3_-)	18 mmol/L	20-32 mmol/L
Sodium ion (Na+)	135 mmol/L	135-145 mmol/L
Potassium (K+)	5.4 mmol/L	3.5-5.2 mmol/L
Serum glucose	5.3 mmol/L	3.0-7.8 mmol/L
Urea	28.3 mmol/L	2.1-7.1 mmol/L
Neutrophils	10.19 x10^9^/L	2.00-8.00 x10^9^/L
Troponin on arrival	93 ng/L	<34 ng/L
Troponin two hours after arrival	93 ng/L	<34 ng/L

The cause of her symptoms remained unclear until a medication transcription error was identified by the ED pharmacist. They identified the patient as at high risk of medication errors, so they telephoned the community pharmacy to obtain an accurate medication history. This unexpectedly revealed a critical error. Her nightly pregabalin dose had mistakenly been increased from 25 mg to 300 mg in the most recently dispensed medication administration aid sachet. The patient reported starting her new medications the night before, so she had likely taken her night-time sachet 18-20 hours prior to presentation. Given her impaired renal clearance, unintentional overdose, and subsequent symptoms, pregabalin toxicity was diagnosed. A differential of uraemic encephalopathy was also considered, but thought unlikely due to her regular community haemodialysis, tolerance of similar levels of serum urea previously, and lack of an obvious precipitant. The patient underwent urgent inpatient haemodialysis, resulting in marked clinical improvement over three sessions, and further doses of pregabalin were withheld.

## Discussion

This report underscores the risk of gabapentinoid toxicity in patients with kidney failure and highlights the importance of careful and timely medication reconciliation in the ED. The ED pharmacist's involvement in this case led to rapid diagnosis and treatment, highlighting their crucial role in patient safety. The case described here can be contrasted with previous case reports, in which gabapentinoids have been identified relatively late as the cause of symptoms. This resulted in further doses being given after symptom onset, and additional patient harm, including intensive care admission for vasopressor support [3,6].

Pregabalin toxicity can mimic serious neurological conditions such as encephalopathy [6], emphasizing the need for a thorough assessment of medication history in any new, unexplained neurological presentation. Given the prolonged course of symptoms and need for dialysis to treat, this presentation would be classified as a grade two, or 'moderate poisoning', in the Poisoning Severity Score [7].

Pregabalin is highly water-soluble and is primarily distributed throughout total body water. It is not bound to plasma proteins and has a molecular weight of 159.23 Da. These properties make it possible to be removed by dialysis [1,3,8]. The improvement in the patients' clinical syndrome after dialysis is in line with the expected dialysable pharmacokinetics, and the response described in previous case reports [3,4].

The temporal association of the increased pregabalin dose with symptoms in a patient at risk for drug accumulation, lack of alternative precipitant, and improvement with dialysis presents a strong case for pregabalin toxicity as a cause of this presentation. It was not felt to be necessary or appropriate to "rechallenge" the patient with a similar dose. Because of this, the presentation is classified as a 'probable/likely' rather than 'certain' adverse drug reaction by the World Health Organization, Uppsala Monitoring Centre Case Causality Assessment [9].

## Conclusions

Gabapentinoid toxicity should be considered in a patient with kidney failure or acute kidney injury who presents with new, unexplained neurological symptoms. Thorough medication reconciliation, in this case done by a dedicated ED pharmacist, is essential to identify such incidents. Dialysis was an effective treatment in this case.
